# Effect of Latex Purification and Accelerator Types on Rubber Allergens Prevalent in Sulphur Prevulcanized Natural Rubber Latex: Potential Application for Allergy-Free Natural Rubber Gloves

**DOI:** 10.3390/polym14214679

**Published:** 2022-11-02

**Authors:** Porntip Rojruthai, Jitladda Sakdapipanich, Jinjutha Wiriyanantawong, Chee-Cheong Ho, Naesinee Chaiear

**Affiliations:** 1 Division of Chemical Industrial Process and Environment, Faculty of Science, Energy and Environment, King Mongkut’s University of Technology North Bangkok, Rayong 21120, Thailand; 2 Department of Chemistry and Center of Excellence for Innovation in Chemistry (PERCH-CIC), Faculty of Science, Mahidol University, Salaya Campus, Phutthamonthon, Nakhon Pathom 73170, Thailand; 3 Sungai Long Campus, University Tunku Abdul Rahman, Cheras Kajang 43000, Malaysia; 4 Department of Community, Family and Occupational Medicine, Faculty of Medicine, Khon Kaen University, Khon Kaen 40002, Thailand

**Keywords:** purified NR latex, saponified NR latex, Type I protein allergy, Type IV chemical allergy, allergenic organic accelerators, allergy-free NR latex, allergy-free NR glove

## Abstract

Natural rubber (NR) gloves manufactured from NR latex are widely utilized in various applications as a personal protective device due to their exceptional barrier characteristics in infection control. However, the use of NR gloves was associated with concerns on NR protein allergy. With comprehensive leaching procedures now a common practice in NR latex glove factories to eliminate latent rubber proteins and chemical allergens, occurrences and complaints of protein allergy from medical glove users have decreased drastically over the past two decades. The present work aims to eliminate further the residual rubber allergens in NR latex through effective purification of the NR latex and compounding the thus purified latex with an established formulation for allergy-free NR for glove applications. NR latex was purified by deproteinization and saponification, respectively. Several analytical techniques were used to verify rubber allergens eliminated in the purified latexes. Saponified NR (SPNR) latex was the purified NR latex of choice since it is devoid of allergenic proteins and poses the lowest risk of Type I allergy. The purified NR latex was compounded with zinc diethyldithiocarbamate (ZDEC), zinc dibutyldithiocarbamate (ZDBC), and zinc 2-mercaptobenzothiazole (ZMBT), respectively, for glove dipping. Among the investigated accelerators, only ZDBC was not detected in the artificial sweat that came into contact with the dipped articles. Thus, it is deduced that ZDBC poses the lowest risk of Type IV allergy to consumers. Additionally, the morphological and physical properties of dipped articles were assessed. It was revealed that the dipped film from the SPNR latex compounded with ZDBC provided thinner and less yellow products with a more uniform internal structure and a tensile strength comparable to those of commercial NR gloves.

## 1. Introduction

An increase in awareness of healthcare and self-protection against highly transmissible diseases is gaining importance nowadays due to the outbreak of coronavirus disease (COVID-19). This has spurred the use of personal protective equipment (PPE), particularly medical gloves, as the first-line protection against this highly infectious disease. Natural rubber (NR) gloves, made from *Hevea brasiliensis* latex, are recognized for their excellent infection control performance based on their superior barrier properties, excellent durability, high tear resistance, good elasticity, and tactile sensitivity [1,2]. Commercial ammoniated NR latex concentrate is a feedstock that can be compounded with many rubber chemicals and latex additives through a process known as vulcanization to convert it into elastic and mechanically strong rubber products such as gloves by dipping. However, naturally occurring rubber proteins in NR latex and some rubber chemicals used in compounding are known allergens. Residuals of these, if not removed from the dipped products, can cause Type I protein and Type IV chemical allergic reactions. Hence the production of NR gloves from compounded latex by dipping process necessitates a rigorous water-leaching step to remove these residual materials to ensure minimal allergic reaction and safe use of the products [3,4].

NR latex contains polyisoprene and nonrubber constituents such as lipids, proteins, carbohydrates, and inorganic compounds [5]. Although NR has outstanding mechanical properties, it is known to contain residual allergenic proteins in latex. Previous studies have shown that various treatments can remove rubber proteins from latex, particularly those on the rubber particles, e.g., enzymatic deproteinization, surfactant washing, saponification, and urea treatments [6,7,8,9,10,11]. Protein removal by urea is an ideal method for industrial applications as it is simple, can be performed at ambient temperature and takes a shorter time than enzymatic treatment [12]. The denaturation of proteins by urea can be described mainly by two different molecular mechanisms. First is direct interaction, and the commonly accepted mechanism is that urea forms strong bonds with the protein and weakens the intermolecular bonds leading to proteins unfolding [13,14,15,16,17]. The urea and water can then access the interior of the protein molecule. The second is an indirect interaction in which urea denatures proteins indirectly by changing the water structure [16,17]. This mitigates the hydrophobic interaction and encourages the solvation of hydrophobic groups. Nevertheless, the mechanism of protein denaturation is far from completely resolved.

Amongst the rubber chemicals and latex additives used in manufacturing NR and synthetic rubber gloves by dipping are stabilizers, vulcanizing agents, accelerators for vulcanization, and antioxidants. Sulphur is widely used as a vulcanizing agent to form crosslinks between the rubber chains in the vulcanization, improving rubber elasticity and mechanical properties. Accelerators such as thiurams, dithiocarbamates, thiazoles, guanidines, and thioureas used in sulphur-vulcanizing systems to increase the speed of vulcanization and improve properties of rubber vulcanizates are known as Type IV allergens [18]. To a lesser extent, excessive residual antioxidants (such as monobenzylether of hydroquinone and p-phenylenediamine) in glove products can also cause Type IV allergies [19,20,21]. Thiurams were mainly allergens detected frequently by patch testing [3]. Tetramethylthiuram disulfide (TMTD) and tetraethylthiuram disulfide (TETD) were reported to possess high allergenic potency in a modified local lymph node assay on the same level as zinc diethyldithiocarbamate (ZDEC) [22]. These thiurams have been replaced with dithiocarbamates and mercaptobenzothiazole derivatives in rubber glove manufacturing since around 2000 [23]. In 2018, it was reported that carbamates were the most common accelerator used in medical examination and surgical gloves at 90.5%, compared with only 5.8% for thiurams of the gloves from 8 major glove manufacturers within the United States [24]. In addition, tetrabuthylthiuram disulfide (TBTD), zinc dibutyldithiocarbamate (ZDBC), and zinc mercaptobenzothiazole (ZMBT), carbamates, and benzothiazoles were recommended for use in NR latex products owing to their low or no sensitizing activity [22]. Among the various extraction mediums such as phosphate buffer, acetone, acetonitrile, and artificial sweat [21,25] employed to determine residual chemicals released from latex products, it was found that extraction with artificial sweat is a useful technique to evaluate the allergenicity of the residual chemical in gloves [23]. Among the dithiocarbamates, it was reported that the amount of ZDEC released from NR latex vulcanizates into artificial sweat was much higher than that of ZDBC [26]. The extent of ZDEC released depended on the amount of ZDEC in formulation and storage time of vulcanizates.

Certain rubber proteins from *Hevea* latex have been reported to trigger Type I immune responses from sensitized individuals [3,18,27,28,29]. Fifteen latex proteins, from Hev b 1 to Hev b 15, were officially listed as NR latex allergens by the International Union of Immunological Societies (IUIS). The detectable allergens in NR gloves were Hev b 1, 2, 3, 5, 6.01, and 6.02 [30,31,32]. Nevertheless, Hev b 13 could be detected in some glove samples [32]. These allergens are present in a different fraction of NR latex after centrifugation, i.e., rubber particles, C-serum, and bottom fraction. Hev b 1 (14 kDa), namely rubber elongation factor (REF), and Hev b 3, namely small rubber particle proteins (SRPP), which are major latex allergens, are hydrophobic proteins bound to rubber particles [33,34,35]. They were presumed to be involved in rubber biosynthesis. Hev b 5 (acidic latex protein) is from the cytoplasmic C-serum. Hev b 2 (β-1,3-glucanases), Hev b 6.01 (Prohevein), Hev b 6.02 (Hevein), and Hev b 13 (Esterase) are found in the serum obtained from the bottom fraction (B-serum) containing lutoids, which are acidic and osmosensitive organelles containing many enzymes [33,35].

The present investigation aims to address these two issues, first by removing the protein (allergens) at the source as much as possible by deproteinization via urea treatment and saponification of the latex. Secondly, the purified latex containing low or no allergenic proteins was compounded with a formulation known for allergy-free dipped products using three different accelerator types in a sulphur-accelerated vulcanization system. The presence of allergenic proteins in the purified NR latex was evaluated and verified. The types of residual accelerators that migrated into artificial sweat in contact with the dipped gloves were elucidated by High-Performance Liquid Chromatography (HPLC). The morphology and physical properties of the dipped gloves were also determined and compared with those of commercial NR gloves. From these experimental findings, a strategy to select a purification method for effective allergenic protein removal from NR latex formulated with allergy-free accelerators to produce gloves with a low risk of Type I and Type IV allergies can be mapped out.

## 2. Materials and Methods

### 2.1. Materials

Field natural rubber (FNR) latex from *Hevea* tree clone RRIT 251 and commercial high-ammonia concentrated natural rubber (CNR) latex sourced from Thai Rubber Latex Group Public Company Limited (Samut Prakan, Thailand). FNR and CNR latexes were preserved with an ammonia solution of at least 0.6% *w/w* of latex. CNR latex is used as control samples. Powdered examination gloves manufactured from NR latex were utilized for comparison with dipped gloves prepared from other compounded latexes. Acetone and methanol (HPLC grade) were supplied by Honeywell and RCI Labscan, respectively. The chemicals for latex compounding, i.e., sulphur, ZDEC, ZDBC, ZMBT, zinc oxide, potassium laurate and Wingstay^®^ L, were commercial grade. Other chemical reagents, i.e., urea, sodium dodecyl sulfate (SDS), Triton^®^ X-100, ammonia, sodium hydroxide, potassium hydroxide, sodium chloride and standard proteins, were analytical grade.

### 2.2. Methods

#### 2.2.1. Preparation of Purified Latex

Deproteinized NR (DPNR) latex was prepared using urea as a protein denaturant. FNR latex was diluted by distilled water to 30% total solid content (TSC) and treated with 0.1% *w/w* solution of urea in the presence of 1% *w/w* SDS. The mixed latex was stirred for 1 h at room temperature before being centrifuged twice at 4 °C, 13,000 rpm. The cream fraction was further dispersed with distilled water to produce DPNR latex containing 60% TSC.

Saponified NR (SPNR) latex was generated using a saponification process with alkali. The FNR latex was diluted to 30% TSC with distilled water, treated with a 1.5% *w/v* solution of sodium hydroxide in the presence of a 1% *w/v* Triton^®^ X-100 at 70 °C for 3 h, and centrifuged twice at 4 °C, 13,000 rpm. The resulting cream fraction was re-dispersed with distilled water to a concentration of 60% TSC to produce SPNR latex.

#### 2.2.2. Preparation of NR Dipping Product

**Figure 1:**
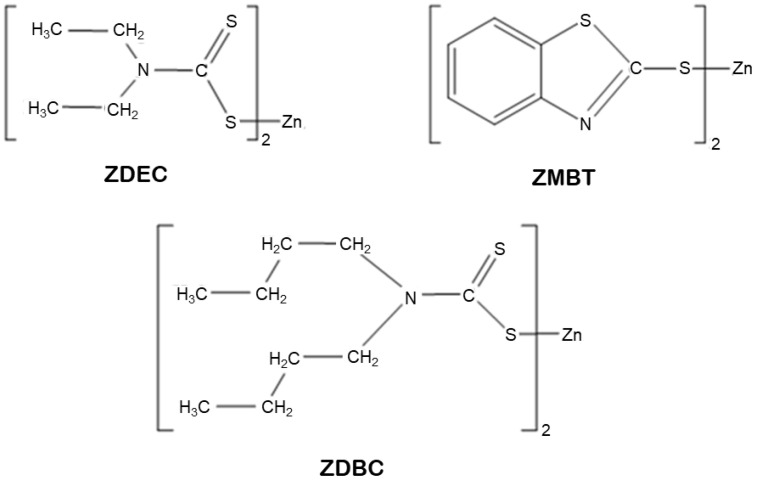
The chemical structures of zinc diethyldithiocarbamate (ZDEC), zinc dibutyldithiocarbamate (ZDBC), and zinc 2-mercaptobenzothiazole (ZMBT).

**Figure 2:**
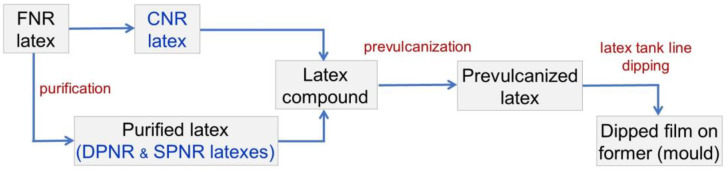
Process for the preparation of NR gloves from the purified latex in this study.

CNR, DPNR and SPNR latexes were treated with different compounding chemicals mentioned in Table 1 and thoroughly agitated at room temperature for 24 h (called maturation) to produce a prevulcanized latex. Figure 1 depicts the molecular structures of the three distinct types of accelerators considered for this study. The heated formers were dipped into a coagulant dispersion of 10% calcium nitrate, 5% calcium carbonate, and 0.05% Triton^®^ X-100 dispersed in distilled water. The coated formers were dried in an oven at 80 °C for 5 min, chilled, dipped into the prevulcanized latex for 15 s, removed, and then postvulcanized by drying in an oven at 80 °C, 100 °C, and 110 °C, respectively, for 10 min in each temperature-step. Leaching was performed by immersing the molds containing the dipped film in 60 °C-distilled water for 10 min while stirring. The dipped product was stripped from the former by applying calcium carbonate powder to the films. The process for preparing NR gloves from the purified latex in this study is depicted in Figure 2.

#### 2.2.3. Extraction of Proteins and Residual Accelerators

Using the SDS-PAGE technique, allergenic protein patterns were found in both pure and pre-vulcanized NR latexes [32]. Briefly, the serum fraction of the latexes generated in the preceding step was suspended in the lysis buffer solution. Proteins were isolated and precipitated using acetone/methanol co-solvent. On the other hand, the extractable proteins from the dried NR samples were stirred into SDS aqueous solution for 12 h then the proteins were precipitated by a co-solvent system. The residual accelerators in the dipped glove were removed with synthetic sweat at pH 6.5 for 24 h at 37 °C [25].

### 2.3. Characterizations

#### 2.3.1. Analysis of Purified NR and Extractable Proteins

The functional groups of the purified NR latexes were identified using a JASCO FT/IR-4100 Fourier-transform infrared (FTIR) spectrometer (JASCO, Tokyo, Japan). The latexes were dried for 12 h at 40 °C in a vacuum oven to yield the dry NR. Before being scanned in the FTIR spectrometer, rubber samples were prepared by depositing solutions of dried NR (1% *w/v* in chloroform) on a KBr plate and then drying with nitrogen gas. Each spectrum was collected using the transmission mode and an average of 100 scans.

The quantities of proteins extracted from each sample were determined using Bradford’s assay after solubilization in lysis buffer [37]. The protein mixtures from both the latex serum and the dry rubber sample were subsequently analyzed by the SDS-PAGE technique. At a constant voltage of 120 V, each protein sample was separated on a 12.5% polyacrylamide separating gel and a 5% polyacrylamide stacking gel. After electrophoresis, the gel was stained for 24 h with colloidal Coomassie blue G-250 for inspection.

#### 2.3.2. Analysis of Residual Accelerators

Artificial sweat extracts of residual accelerators from the dipped gloves were transformed into copper complexes for HPLC analysis [25] (Dionex Ultimate 3000, Thermo Fischer Scientific, Waltham, MA, USA) on a reversed-phase column (Acclaim RSLC C18, 2 μm, 120 Å, 2.1 × 100 mm, Thermo Fisher Scientific, Waltham, MA, USA). The column was eluted for 10 min using a 90:10 (*v/v*) mixture of methanol and water as a mobile phase. The eluent was pumped and monitored at a flow rate of 0.5 mL/min at 435 nm. The peak area was utilized to estimate the concentration of the accelerator and the retention duration for identification. As standards, stock solutions of the copper complex containing actual amounts of ZDEC, ZDBC, and ZMBT were prepared with 1% *w/v* of the accelerator in dichloromethane. These were subjected to a series of dilutions to generate copper complexes. From triplicate injections, the average peak area of the corresponding copper complex at their retention time was computed to establish the calibration curve.

#### 2.3.3. Physical Properties and Morphology of the NR Gloves

Kimwipes^®^ were used to remove excess calcium carbonate powder from rubber-dipped samples, which were then cut into the desired size. An ASTM E313-compliant benchtop grating spectrophotometer-YS 6010 model (3nh^®^, Shenzhen, China) was operated in transmission mode at 0° with an illuminant D65 light source to assess the cut rubber film samples’ yellowness index (YI).

The internal morphology of each rubber sample was analyzed using a field emission scanning electron microscope (FE-SEM), SU-8010 (Hitachi, Tokyo, Japan), with 10 kV accelerating voltage. The samples were generated using a cryogenic fracturing procedure in liquid nitrogen in which the frozen sample was fractured. After drying the samples, the cracked surface was sputtered and coated with a thin conductive layer of palladium (Pd) to observe the internal structure.

The sample of the dipped finger cots, prepared as a representative of NR gloves, was cut into a rubber band shape before being tested with a tensile tester (INSTRON model 5566, Instron, Norwood, MA, USA), according to Thailand Industrial Standard TIS 2725−2559. The sample was clamped to a tensile tester with a 1 kN load cell and stretched at a crosshead speed of 500 mm/min until the break. The tensile parameters of the samples (tensile modulus, tensile strength, and elongation at break) were measured as the average of five measurements.

The crosslink density of the vulcanized sample was measured using the solvent swelling method. The sample was cut into a 1 cm^3^ shape and then immersed in 100 mL of toluene at room temperature for a week. The weight of the sample before and after swelling was obtained to determine crosslink density according to the Flory-Rehner equation (Equation (1)) [38].

(1) $$v=-(\ln(1-V_{r}^{0})+V_{r}^{0}+\chi V_{r}^{0^{2}})/2\rho V_{0}\left(V_{r}^{0^{\frac{1}{3}}}-\frac{V_{r}^{0}}{2}\right)$$

where $V_{r}^{0}$ is the volume fraction of the vulcanized rubber. χ is the Huggins interaction constant of gum NR with toluene which is 0.39. $V_{0}$ is the molar volume of the solvent used; 106.9 cm^3^/mol for toluene. The $V_{r}^{0}$ was obtained using Equation (2)

(2) $$V_{r}^{0}=\frac{1}{\left(\frac{\rho_{r}}{\rho_{s}}\right)\left(\frac{W_{s}-W_{u}}{W_{u}}\right)}+1$$

where $W_{u}$ and $W_{s}$ are the weights of the unswollen and swollen rubber samples at equilibrium, respectively. $\rho_{r}$ and $\rho_{s}$ are the densities of NR (0.930 g/cm^3^) and toluene (0.886 g/cm^3^).

## 3. Results and Discussion

### 3.1. Characterization of Purified Rubber by FTIR and SDS-PAGE

**Figure 3:**
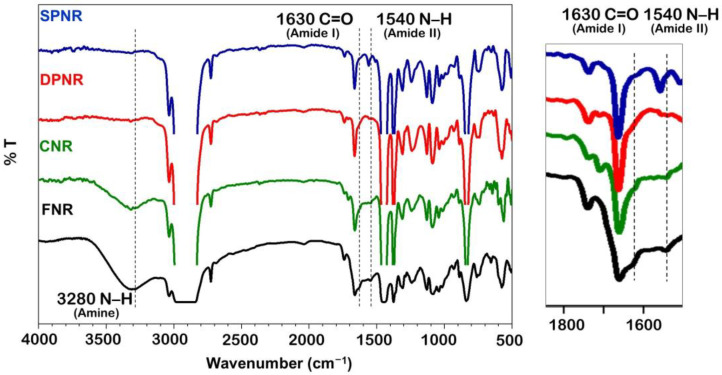
FTIR spectra of FNR, CNR, DPNR, and SPNR latexes.

Figure 3 shows the FTIR spectra of the purified NR to monitor the change in their protein contents after purification. From the spectra of FNR, CNR, and purified NRs (DPNR and SPNR), it can be determined that the N-H bond at wavenumber 3280 cm^−1^ corresponds to the amino group in proteins is absent in the purified NR sample. Additionally, it was discovered that the purified NR samples lacked the two peaks at 1630 and 1541 cm^−1^. These two peaks correspond to the C=O bond of the amide group and the N-H bond of the amide group, respectively, in the protein structure [39]. Hydrolysis of the amide in proteins and polypeptides during saponification causes a shift in the N-H bond of amide, as shown by the presence of an additional peak in the SPNR rubber at around 1560 cm^−1^ [40]. It also indicates that the SPNR contains some residual nitrogenous material, including proteins and polypeptides [40]. The nitrogen contents of FNR, CNR, DPNR, and SPNR are determined to be 0.813, 0.399, 0.063, and 0.033 %, respectively, which compare favorably to those reported in the literature [40].

**Figure 4:**
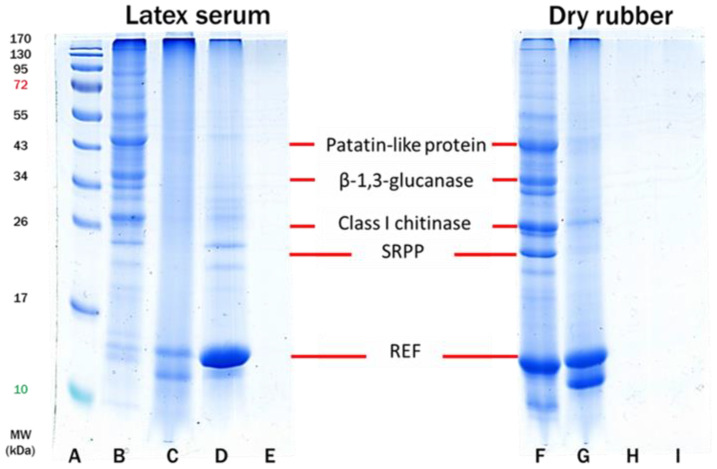
SDS-PAGE profiles of proteins extracted from latex serum (Lanes: **B**–**E**) and dry rubber sheet (Lanes **F**–**I**) where Lane (**A**): protein marker; Lane (**B**): FNR latex; Lane (**C**): CNR latex; Lane (**D**): DPNR latex; Lane (**E**): SPNR latex; Lane (**F**): dry FNR; Lane (**G**): dry CNR; Lane (**H**): dry DPNR and Lane (**I**): dry SPNR.

As shown in Figure 4, the SDS-PAGE technique was also used to characterize and validate the presence of the types of proteins in latex serum and cast-dried purified rubbers. All the allergens found in Figure 4 may be extracted from latex serum and dried rubber [33,35,37,41,42]. In the latex serum of DPNR, the protein band at around 170 kDa had diminished, whereas those at approximately 15 kDa had increased and become conspicuous. This may suggest that the large molecular weight proteins of 170 kDa unfolded into smaller units upon contact with urea and were subsequently eliminated by centrifugation [43,44,45]. After removing the original intact proteins from the NR latex particles during the deproteinization of FNR latex with urea, the powerful surfactant SDS was applied to stabilize the latex (mainly the REF proteins from the large rubber particles). After centrifugation of the urea-treated latex, nearly all of the dislodged REF proteins will be in the serum, with very few remaining on the large rubber particles in the DPNR latex cream.

Consequently, the strong band at 15 kDa for DPNR (Lane D in Figure 4) corresponds to the residual REF proteins in the latex serum. It is assumed that the cream fraction of the DPNR latex, after drying at high temperature and casting on a glass plate, would contain no extractable proteins, as centrifugation would have eliminated all proteins displaced by SDS (Lane H in Figure 4). REF proteins, highly allergenic proteins of NR latex, are strongly linked to large rubber particles [46] and hence difficult to remove unless they have been replaced by a more potent surfactant such as SDS utilized in the denaturation process of proteins by urea.

Even when a high amount of latex serum and dry rubber samples were employed for extraction, no detectable proteins could be extracted from the DPNR and SPNR samples. This result indicates that the purified NR, particularly the SPNR, may be effectively prepared without allergenic proteins.

### 3.2. Effect of Accelerator Types on Allergenic Proteins of Purified NR

**Figure 5:**
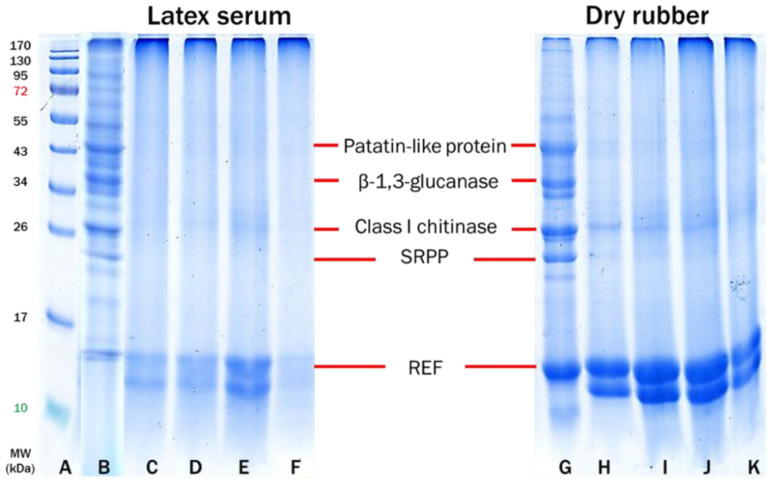
SDS-PAGE profiles of extracted proteins from latex serum (Lanes **B**–**F**) and dry rubber sheet (Lanes **G**–**K**) from CNR vulcanized with the accelerator type or vulcanized CNR (VC) as indicated: Lane (**A**): protein marker; Lane (**B**): FNR latex; Lane (**C**): CNR latex; Lane (**D**): VC-ZDEC latex; Lane (**E**): VC-ZDBC latex; Lane (**F**): VC-ZMBT latex; Lane (**G**): dry FNR; Lane (**H**): dry CNR; Lane (**I**): dry VC-ZDEC; Lane (**J**): dry VC-ZDBC and Lane (**K**): dry VC-ZMBT.

The CNR and purified latexes (DPNR and SPNR) were compounded using ZDEC, ZDBC, and ZMBT as organic accelerators. The rubber proteins were extracted from the compounded latexes and the vulcanized dried rubber samples. Latex serum from prevulcanized CNR (VC) latexes (Lanes: D, E, F) and dry rubber from postvulcanization of the latexes (Lanes: I, J, K) demonstrate (Figure 5) the presence of known allergenic proteins known to be present in *Hevea.* In addition, the allergenic REF proteins (14 kDa) are notably abundant in all tested samples, regardless of the types of accelerators employed. The REF proteins are also found in the latex serum and dry rubber film extracts from the unvulcanized and vulcanized CNR samples (Lanes: C and H). This can be interpreted to mean that allergenic proteins are always present in the dipped products (gloves) when CNR latex is used for compounding/vulcanization, regardless of whether the organic accelerators are utilized.

**Figure 6:**
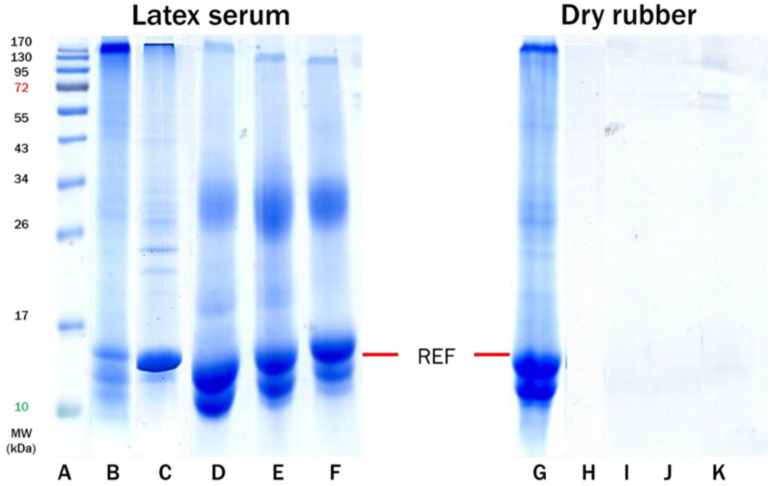
SDS-PAGE profiles of proteins extracted from latex serum (Lanes **B**–**F**) and dry rubber films (Lanes **G**–**K**) from DPNR samples vulcanized with the accelerator type or vulcanized DPNR (VD) as indicated: Lane (**A**): protein marker; Lane (**B**): CNR latex; Lane (**C**): DPNR latex; Lane (**D**): VD-ZDEC latex; Lane (**E**): VD-ZDBC latex; Lane (**F**): VD-ZMBT latex; Lane (**G**): dry CNR; Lane (**H**): dry DPNR; Lane (**I**): dry VD-ZDEC; Lane (**J**): dry VD-ZDBC; and Lane (**K**): dry VD-ZMBT.

For the DPNR samples, it could be seen from Figure 6 that there was a high-molecular-weight protein band around 170 kDa and a prominent REF protein band at 14.6 kD observed in the serum fraction of unvulcanized DPNR latex extract (Lane C) as explained earlier. In contrast, no extractable REF proteins were found in the dried DPNR (Lane H). However, as expected, prominent REF protein bands were observed in the serum of all prevulcanized DPNR (VD) latexes containing the different accelerators (Lanes: D, E, F). When the prevulcanized latexes with different accelerators were cast and dried during the postvulcanization step, no water-soluble proteins were extracted from the samples (Lanes: I, J, K). It is deduced that no water-soluble allergenic proteins were extracted when purified DPNR latex was compounded with any of the three accelerators chosen. This implies that DPNR latex can be used to produce gloves without water-soluble allergenic proteins.

**Figure 7:**
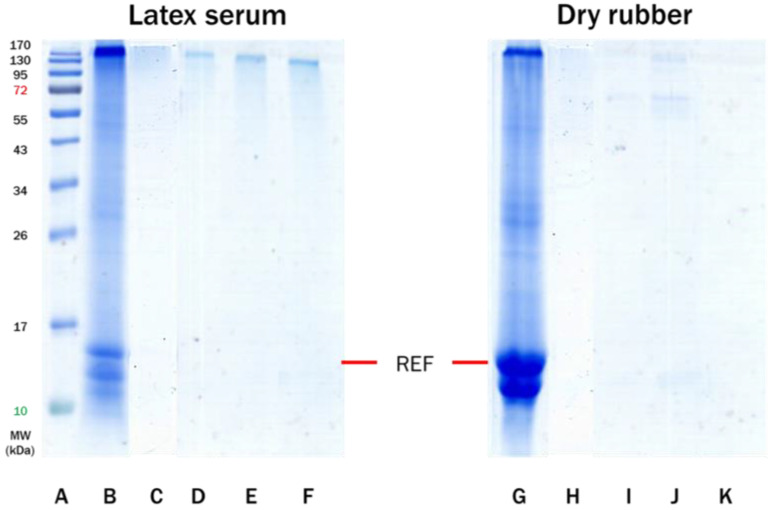
SDS-PAGE profiles of proteins extracted from latex serum (Lanes **B**–**F**) and dry rubber sheet (Lanes **G**–**K**) from SPNR samples vulcanized with the accelerator type or vulcanized SPNR (VS) as indicated: Lane (**A**): protein marker, Lane (**B**): CNR latex; Lane (**C**): SPNR latex; Lane (**D**): VS-ZDEC latex; Lane (**E**): VS-ZDBC latex; Lane (**F**): VS-ZMBT latex; Lane (**G**): dry CNR; Lane (**H**): dry SPNR; Lane (**I**): dry VS-ZDEC; Lane (**J**): dry VS-ZDBC; and Lane (**K**): dry VS-ZMBT.

For the SPNR sample, the FNR latex was treated with NaOH at a high temperature in base-catalyzed hydrolysis of proteins and lipids in NR, resulting in a decrease in protein and lipid concentrations [42,47]. Based on these data, it was evident from Figure 7 that there was no protein extractable from either pre-vulcanized SPNR (VS) latex serum or dry rubber of any vulcanized SPNR, regardless of the accelerator utilized. The absence of extractable proteins from the SPNR latex and SPNR dipped films demonstrates that this purified latex is free of allergenic proteins.

### 3.3. Effect of Purified NR and Accelerator Types on the Migration of Residual Accelerators from Glove to Artificial Sweat

The present study examined the migration of three compounding accelerators from dipped films to artificial sweat. This is one of the most significant analyses of glove wearers’ allergic responses to substances. ZDEC and ZDBC are the main dithiocarbamate accelerators with an extremely rapid vulcanized rate. The difference between ZDEC and ZDBC is the substituent alkyl group, which is ethyl for ZDEC and butyl for ZDBC (Figure 1). ZMBT, a thiazole accelerator, is commonly employed as a secondary accelerator in conjunction with dithiocarbamate in rubber latex vulcanization.

**Figure 8:**
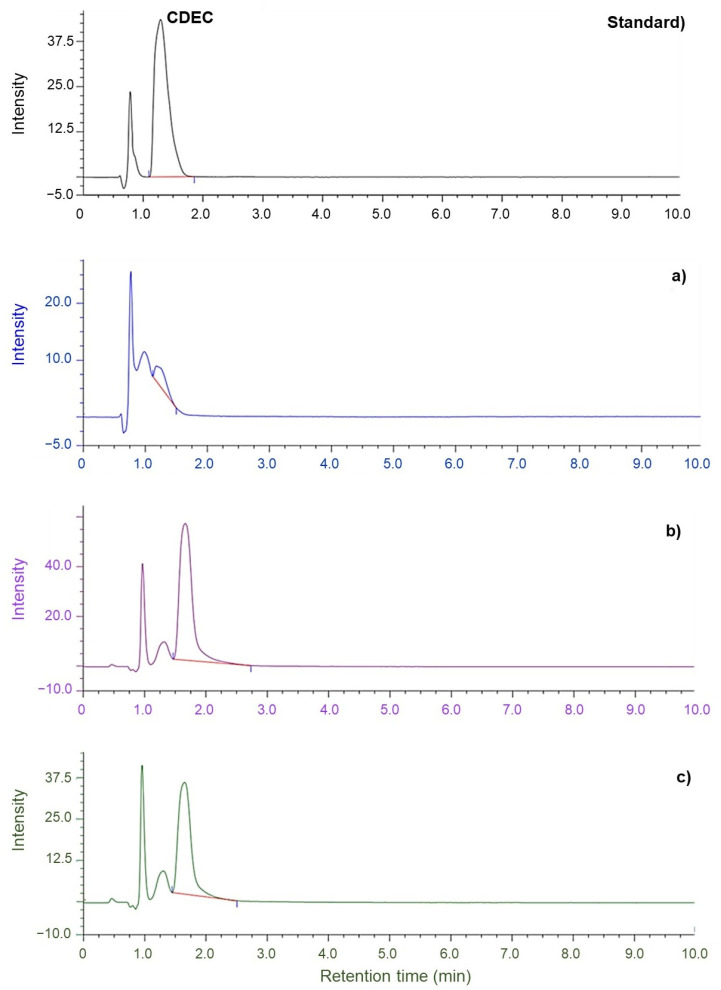
Chromatograms of (standard): CDEC and artificial sweat solution containing extracted accelerators from gloves made from (**a**): VC-ZDEC latex, (**b**): VD-ZDEC latex, and (**c**): VS-ZDEC latex after converting to CDEC, followed by determination using HPLC. (VC: vulcanized CNR, VD: vulcanized DPNR, and VS: vulcanized SPNR).

**Figure 9:**
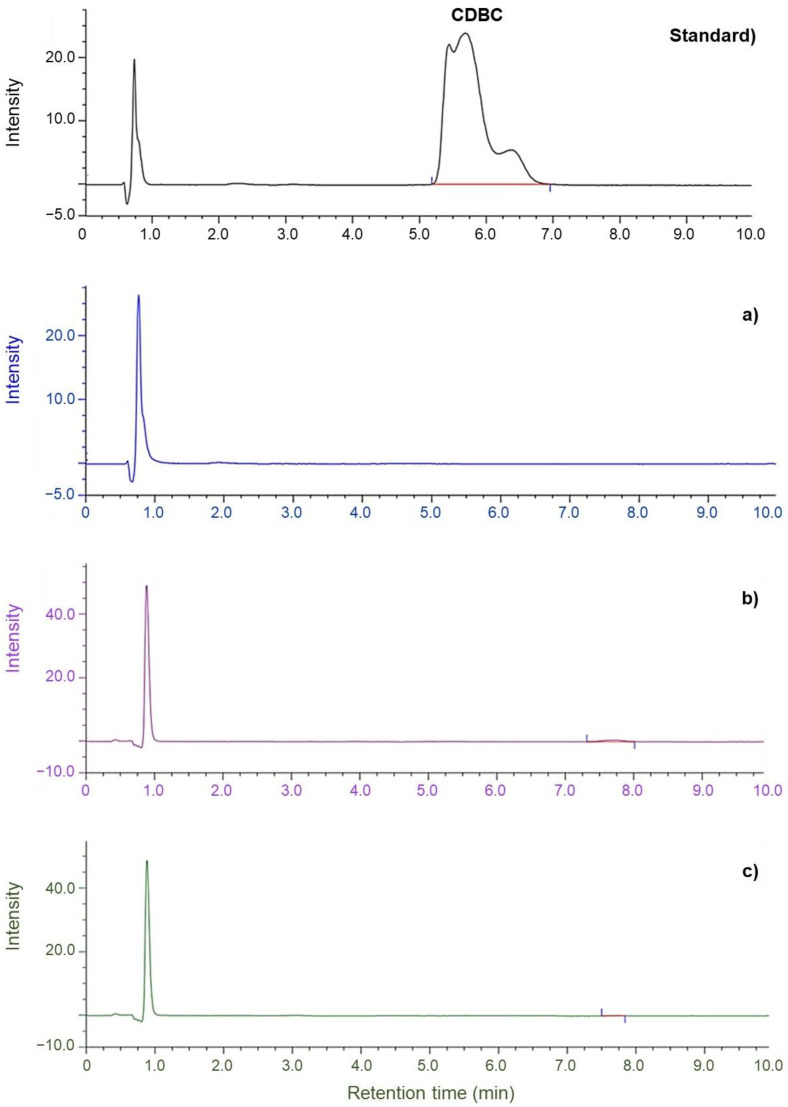
Chromatograms of (standard): CDBC and artificial sweat solution containing extracted accelerators from gloves made from (**a**): VC-ZDBC latex, (**b**): VD-ZDBC latex, and (**c**): VS-ZDBC latex, respectively, after converting to CDBC followed by determination using HPLC. (VC: vulcanized CNR, VD: vulcanized DPNR, and VS: vulcanized SPNR).

Figure 8 and Figure 9 depict the chromatograms of ZDEC and ZDBC recovered from the NR-dipped gloves (produced from the CNR, DPNR, and SPNR latexes) using artificial sweat. The copper complex of ZDEC (CDEC) was initially prepared as standards and identified as a typical sharp peak at a retention time of 1.3 min at the peak of the mobile phase in HPLC. In the artificial sweat samples derived from VC-ZDEC, VD-ZDEC, and VS-ZDEC, it was evident from the retention time data in Figure 8a–c that the CDEC was detected in the dipped gloves prepared from all latex types. Figure 9a–c demonstrates that the CDBC, detected at a retention time of 6.2 min, was absent from all artificial sweat extracted from NR-dipped gloves.

Based on the acquired calibration curve of CDEC, the peak areas at 1.3 min retention time in the chromatogram shown in Figure 8a–c were used to quantify the quantity of accelerator released into the artificial sweat from each dipped glove sample. So far, ZDEC peak was found in all from the dipped glove samples could be quantified from the artificial sweat analysis. However, ZDBC was not detectable in the artificial sweat used for the extractions of gloves with all the three different accelerators, Figure 9a–c. This may be attributed to the fact that ZDBC, which has a larger hydrophobic butyl group than ZDEC, has a considerably maller water solubility than ZDEC and is hence less soluble in artificial sweat. The migration of the accelerator from the dipped glove to the artificial sweat depends not only on the water solubility of the utilized accelerators but also on the concentration and rate of migration [26].

Consequently, the selection of accelerators for latex compounding is a crucial concern. Thus, using ZDBC in vulcanization would provide gloves with a far less residual extractable accelerator that is more user-friendly. Comparatively, the predicted migration of ZDEC from the dipped glove produced from DPNR and SPNR latexes are 88 µg/g and 59 µg/g, respectively (Figure 8b,c: chromatograms VD-ZDEC and VS-ZDEC, respectively).

Finally, it was discovered that ZMBT, a thiazole accelerator, was not particularly soluble in dichloromethane, resulting in the formation of the turbid solution, including solid sediment, when copper ZMBT standard solutions were prepared using the same procedure as ZDEC and ZDBC. This rendered the calibration and the artificial sweat extraction experiment invalid. Therefore, no information on the migration of this accelerator from the dipped glove was available.

### 3.4. Properties of Products

#### 3.4.1. Product Appearance

**Figure 10:**
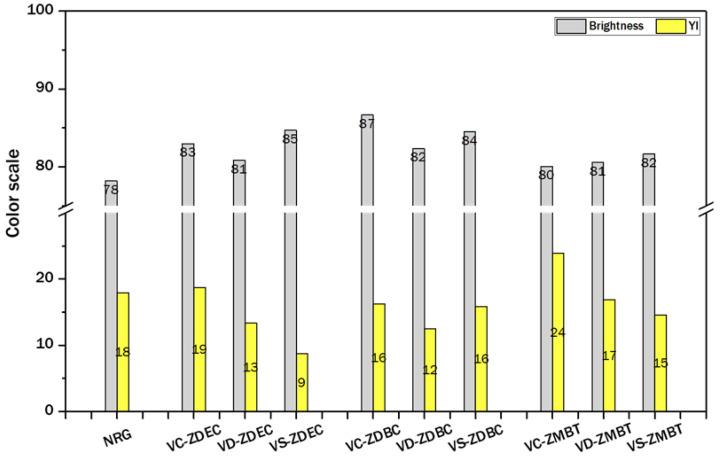
Brightness and yellowness indices were obtained from NR gloves prepared from various prevulcanized latex types (VC: vulcanized CNR, VD: vulcanized DPNR, and VS: vulcanized SPNR), accelerators, and commercial NR latex gloves (NRG).

Figure 10 shows the brightness and yellowness index of NR-dipped glove samples prepared using three different latexes compounded with three different accelerators, as measured by the YS6010 spectrophotometer. The results indicate that the brightness of gloves prepared from DPNR and SPNR latexes falls within the same range as those from the commercial NR glove (NRG in Figure 10). In contrast, the yellowness index of commercial NR gloves is generally higher than those from this work, except for VC-ZMBT gloves. This could be attributed to the effective removal of the residual-colored substances such as carotenoid, unsaturated fatty acid, fatty alcohol, monoglyceride, and polyphenol oxidase from the CNR latex by purification and centrifugation processes, resulting in much lighter colored gloves dipped using the purified latexes of DPNR and SPNR with ZDEC and ZDBC as accelerators [48,49]. This study’s gloves had a thickness of around 0.2 mm, which is within the standard range for commercially available disposal NR gloves.

#### 3.4.2. Morphology of Internal Structure

**Figure 11:**
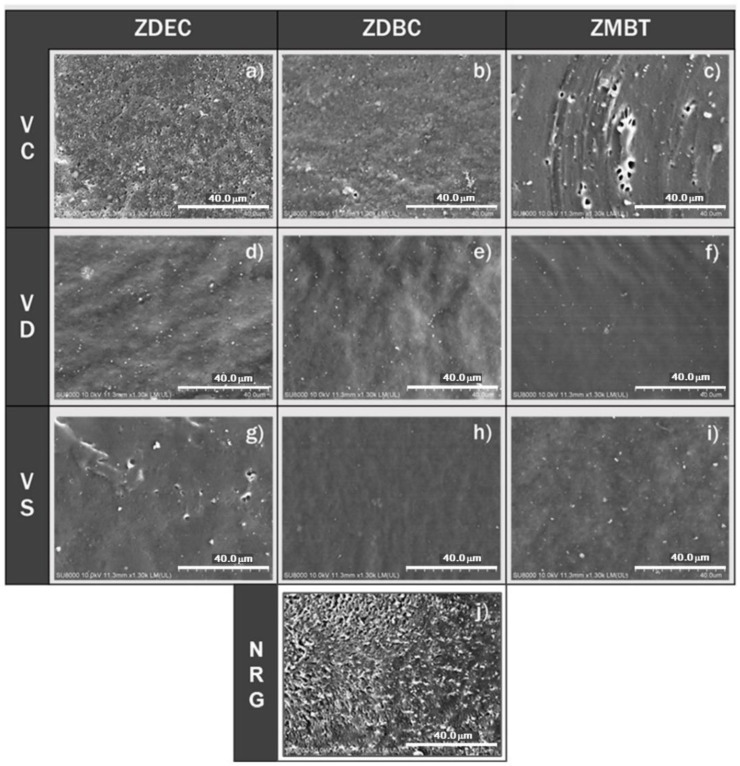
SEM images of freeze-fractured surface (internal structure) of gloves prepared by dipping various prevulcanized latex types (VC: vulcanized CNR, VD: vulcanized DPNR, and VS: vulcanized SPNR) containing various accelerators (ZDEC, ZDBC, and ZMBT), i.e., (**a**): VC-ZDEC, (**b**): VC-ZDBC, (**c**): VC-ZMBT, (**d**): VD-ZDEC, (**e**): VD-ZDBC, (**f**): VD-ZMBT, (**g**): VS-ZDEC, (**h**): VS-ZDBC, and (**i**): VS-ZMBT, and (**j**): commercial NR latex gloves (NRG).

Figure 11 depicts the SEM micrographs of the shattered surface of the gloves that reveal the interior structure of the gloves that were dipped for this investigation. It can be observed that the dipped gloves from the purified DPNR and SPNR latexes are uniform and smooth, with no obvious creation of inner cavities (Figure 11d–i except for Figure 11g). In comparison, films made from CNR latex are not homogeneous, with significant voids (holes) apparent throughout the core, especially in those containing ZDEC and ZMBT (Figure 11a,c). In addition, both large and small voids could be observed (Figure 11c,g). The film from commercial gloves (Figure 11j) is equally heterogeneous, with a rough fractured surface and many cavities within. The uniform and smooth film from the purified DPNR and SPNR latex resulted from the removal of the protein layer surrounding the latex particle that promoted the effective coalescence of latex particles (interparticle crosslinking) after drying.

In contrast, the heterogenous film due to the spherical contour feature of the latex particle in the presence of protein could be observed in the film from the CNR latex and the commercial glove. The small holes in the film made from CNR latex were possibly caused byparticle of residual chemicals breaking off, whereas pinholes from air bubbles trapped during the dipping process. Any known discontinuity or imperfection in the film structure can be converted into glove defects, leading to a subsequent breakdown in barrier characteristics. It has always been known that NR gloves offer higher barrier qualities compared to gloves fabricated from other latexes. This work reveals that gloves produced from DPNR and SPNR latexes compounded with ZDBC have a smooth and homogeneous internal film structure, which is desirable in a barrier material.

#### 3.4.3. Mechanical Properties

**Figure 12:**
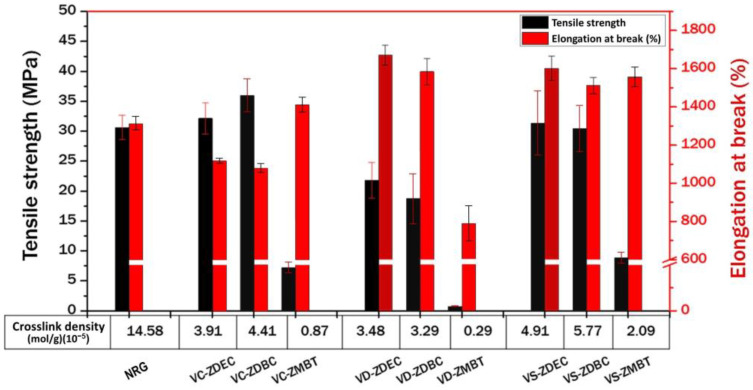
Crosslink density, tensile strength, and elongation-at-break of gloves obtained from various prevulcanized latex types (VC: vulcanized CNR, VD: vulcanized DPNR, and VS: vulcanized SPNR) containing various accelerators (ZDEC, ZDBC, and ZMBT). NRG is commercial NR gloves.

Figure 12 revealed that gloves prepared from the commercial CNR and purified SPNR latexes compounded with ZDEC, ZDBC, and ZMBT exhibited similar vulcanizate qualities of high tensile strength and elongation at break as the commercial NR gloves (NRG). In contrast, gloves prepared from the DPNR latex, which has far fewer residual proteins than the CNR latex and no by-products of lipid decomposition such as SPNR latex, have the lowest crosslink density and tensile strength. The figure demonstrates that ZMBT, a secondary accelerator, generated gloves with the lowest crosslink density and tensile strength when employed alone. It is also noted that the crosslinked density of commercial NR gloves is significantly greater than those produced by hand dipping in the laboratory due to many factors, such as the formulation of the latex compound with different types and contents of chemicals, especially sulphur and accelerator, the prevulcanization condition (temperature and duration time), and production process and condition in glove factories, e.g., drying time and temperature, leaching step, etc. The main factors that affect the crosslink density of latex film have been reported to be sulphur content and prevulcanization temperature [50]. As seen in Table 1, the sulphur content in the formulation for surgical gloves, which are required to be of low elastic modulus, is low; therefore, this was possibly the reason for the lower crosslink density. In addition, the other factors may be caused by the consistency of dipping operating conditions and processes in glove factories and the frequently employed hybrid accelerator system in the commercial production of NR gloves. However, the behavior observed in Figure 12 is sufficiently typical to prove that gloves prepared from the SPNR latex compounded with ZDEC or ZDBC would have equivalent mechanical qualities to those of the commercial NR gloves.

## 4. Conclusions

NR gloves prepared by deproteinization and saponification methods and, subsequently, pre-vulcanized using ZDEC, ZDBC, and ZMBT, respectively, as accelerators were employed to assess the effect of purification and accelerators types on residual rubber allergens in rubber-dipped products. The results demonstrate that only purified SPNR latex is devoid of allergenic proteins in the serum, as verified and confirmed by several analytical techniques. Additionally, no allergenic proteins were found in the artificial sweat extract in contact with the gloves prepared from SPNR latex. When this latex is compounded with ZDEC and ZDBC, respectively, and made into gloves, no ZDBC was found to migrate from the gloves into the artificial sweat in contact with it. The gloves made from purified SPNR latex were also lighter in color (yellowness) and brighter due to the effective removal of residual colored constituents from the CNR latex during purification and centrifugation. SPNR latex also formed a more homogeneous film which means it is better barrier material for glove application as borne out by the physical properties of the glove samples prepared from SPNR latex using the ZDBC accelerator. In conclusion, the present findings indicate that the purified SPNR latex compounded with ZDBC might be used to produce Type I and Type IV allergy-free NR latex gloves with qualities comparable to those of conventional NR gloves. These attributes are anticipated to be enhanced when transferred to a glove manufacturer’s current, cutting-edge commercial production settings.

## Figures and Tables

**Table 1:** Latex compound formulations for sulphur-vulcanized gloves using different NR latexes and accelerators (see Figure 1).

Ingredients	phr *
60% CNR, DPNR, or SPNR latex, respectively	100
20% KOH solution	0.3
20% Potassium laurate solution	0.2
50% Sulphur dispersion	0.5
50% ZDEC, ZDBC, and ZMBT dispersions, respectively	0.75
50% Wingstay^®^ L dispersion	0.5
50% ZnO dispersion	0.25

* The chemical compositions were followed the published formulation [36] in phr (part per hundred rubber).

## Data Availability

Not applicable.
